# Automated estimation of the logistic curve model of cerebral CO_2_ vasomotor reactivity

**DOI:** 10.1007/s10877-026-01466-4

**Published:** 2026-07-07

**Authors:** R. B. Panerai, J. Ince, A. Alshehri, R. H. Clough, T. G. Robinson, J. S. Minhas

**Affiliations:** 1 Cerebral Haemodynanics in Ageing and Stroke Medicine (CHiASM), Division of Cardiovascular Sciences, University of Leicester, Leicester, UK; 2 NIHR Leicester Biomedical Research Centre, Leicester British Heart Foundation Centre of Research Excellence, Glenfield Hospital, Leicester, UK; 3 Emergency Medical Services Department, College of Applied Medical Sciences, Najran University, Najran, Saudi Arabia; 4 Robert Kilpatrick Clinical Sciences Building, University of Leicester, Room 439 Leicesster Royal Infirmary, LE2 7LX Leicester, United Kingdom

**Keywords:** Cerebral autoregulation, Cerebral blood flow, Logistic model, Biological sex, Automated analysis

## Abstract

An automated method is presented to derive the logistic curve model (LCM), to express the simultaneous effects of partial pressure of arterial CO_2_ (PaCO_2_) on cerebral blood flow (CBF) and cerebral autoregulation, without the need for operator assistance. Measurements of middle cerebral artery blood velocity (MCAv, transcranial Doppler), arterial blood pressure (Finometer) and end-tidal CO_2_ (EtCO_2_, capnography) were performed in 106 healthy subjects. Hypercapnia was induced with breathing 5% CO_2_ and hypocapnia was induced with hyperventilation. Dynamic autoregulation was expressed by the Autoregulation Index (ARI). Confidence limits for the fitting error were obtained with a surrogate bootstrap approach for both MCAv and ARI. An algorithm for the gradual removal of outlier values was implemented to identify optimal number of samples that minimises the fitting error. Induction of hyper- and hypocapnia was demonstrated by the range of EtCO_2_ values achieved (28.4 ± 6.0 to 47.7 ± 5.8 mmHg, *p* < 0.001). LCMs meeting confidence limit requirements were obtained for MCAv (*n* = 98/106) and ARI (104/106). Females showed an upwards shift of their LCM for MCAv (*p* = 0.001) compared to males and their ARI model was shifted laterally towards lower values of EtCO_2_ (*p* = 0.0053). Automated identification of the LCM for MCAv and ARI, with rigorous confidence limits of the fitting error is feasible and can identify differences due to biological sex.

## Introduction

The cerebral circulation is highly sensitive to changes in the partial pressure of arterial CO_2_ (PaCO_2_), with hypercapnia leading to vasodilation and hypocapnia having the inverse effect [1]. This response of the cerebral vasculature to changes in CO_2_ is referred to as *CO*_*2*_ *vasomotor reactivity* (VMR), and is regarded as a homeostatic mechanism which automatically adjusts the removal of by-products of metabolism in response to the build up of CO_2_ in cerebral tissues, due to variations in energy demand [2]. In addition to its effect on cerebral blood flow (CBF), mediated by changes in pH, VMR can interact with other cerebrovascular regulatory mechanisms, such as cerebral autoregulation (CA), the ability of vascular smooth muscle to adjust cerebrovascular resistance (CVR) in response to changes in mean arterial pressure (MAP) [2, 3]. CA can be assessed with either static or dynamic modelling methods [4], and both modalities indicate that hypercapnia leads to a less efficient regulatory response to changes in MAP, while hypocapnia increases the performance of CA [1–3, 5–9].

CO_2_ VMR can change due to different physiological conditions, such as posture [10, 11], but also in disease states, such as ischemic stroke [12], carotid artery disease [13], and hypertension [14]. Methodologically, studies of VMR in different physiological or pathological conditions have used a variety of measurement and protocol techniques to induce changes in PaCO_2_, as well as to measure the corresponding response of CBF, often with surrogate approaches such as transcranial Doppler ultrasound (TCD) [15] or magnetic resonance imaging [16]. This methodological diversity has led to a correspondingly varied literature where it is very difficult to make inter-study comparisons or to establish reference values adjusted for age and biological sex. As an example, it is nearly impossible to compare results from studies that used either acetazolamide, breath-holding, fixed inspired CO_2_ in air, rebreathing from a bag, or computerized end-tidal forcing, to induce hypercapnia [1]. Above all, there has not been a standardization of the PaCO_2_ change that should be elicited for calculation of VMR indices, and it is to be expected that a change from 35 to 38 mmHg for example, as usually estimated with end-tidal CO_2_ (EtCO_2_), would lead to different values from say, 40 to 50 mmHg [6, 17].

To address the main limitations highlighted above, we have proposed a more comprehensive approach to the quantification of CO_2_ VMR covering the entire physiological range of PaCO_2_, as estimated with measurements of EtCO_2_ [8, 10, 18]. This approach involves multiple measurements of cerebral blood velocity (CBv), MAP and EtCO_2_, combined with the representation of the effects of CO_2_ using a logistic curve model (LCM). The proposal that the dependence of CBF or CVR on PaCO_2_ follows a logistic curve has been made by previous investigators [17, 19–21]. We have extended their pioneering work, to also include the effects of PaCO_2_ on dynamic CA, as expressed by the autoregulation index (ARI) [4]. The ARI ranges from 0 (absence of CA) to 9 (best CA usually observed). Although initially proposed to model the CBv response to a rapid drop in MAP induced by the sudden release of compressed thigh cuffs [4], it has been demonstrated that it is also feasible to derive estimates of ARI from spontaneous fluctuations in MAP and CBv [22]. Therefore, having the complete dependence of CBv, ARI and other cerebral hemodynamic parameters, such as critical closing pressure and resistance-area product on EtCO_2_, over its entire physiological range, provides a much more complete ‘mapping’ of CO_2_ VMR, as compared to more traditional punctual estimates [8, 10, 18].

One drawback of our approach to obtain estimates of the entire LCM though, has been the need to adopt interactive visual control to minimize the error of model fitting by sequential elimination of outliers [10]. See also https://doi.org/10.6084/m9.figshare.25745430. Although this approach has led to robust results, it is a time-consuming procedure and the subjectivity of visual control by different operators is not desirable. The objective of this article is to describe how we have overcome these limitations by (i) deriving the confidence limits of the LCM fitting error using a surrogate data bootstrap technique and (ii) implementing an automated algorithm to optimize the fitting error, taking the confidence limits into account. Moreover, we also describe the results of the automated procedure on a large set of physiological recordings from healthy subjects to create a pathway to analysis in disease groups.

## Methods

### 2.1 Subjects and measurements

The data for this study were collated from three previous studies by our group, based on the same protocol and physiological measurement procedures performed to uniform standards [8, 10, 18]. Ethical approval for the three studies was granted by the University of Leicester Ethics Committee (References: jm591-c033; 28664-jm591-ls: cardiovascularsciences; 37467-jm591-ls: cardiovascular sciences). The studies were conducted in line with the Declaration of Helsinki (2013) but were not recorded in a publicly accessible database. All participants provided written informed consent. Participants were healthy university staff and students. All participants were over the age of 18 years old. Exclusion criteria included being pregnant or breastfeeding, or any significant history of cardiovascular, neurological, or respiratory disease. Well-controlled chronic hypertension (single anti-hypertensive agent) in otherwise healthy and active participants was accepted. Participants were asked to refrain from strenuous exercise in the 12 h prior to the study, and from caffeine, smoking, alcohol and heavy meals 4 h prior to study commencement.

The protocol for the three previous studies consisted of measurements in the supine position, with two distinct recordings, one to induce hypercapnia and the second to induce hypocapnia. Both recordings started with a baseline period of poikilocapnia at rest. In the first recording, 2 min of baseline was followed by two minutes of breathing 5% CO_2_ in air through a mask, for induction of hypercapnia, then a further two minutes of breathing at rest. After an additional five minutes of rest, the second recording consisted of 90 s of poikilocapnia, three minutes of paced hyperventilation, and a further 90 s of breathing at rest. For the hyperventilation procedure, participants were asked to breathe in time with a metronome (KORG MA-30) at a rate of 20 breaths per minute. Participants were instructed to breathe through their nose and to follow the frequency given by the metronome. Metronome pacing has been demonstrated to be effective at inducing and maintaining hypocapnia [7, 23]. The start and end of the exposure to 5% CO_2_ or hyperventilation were marked using an electric switch.

All three studies were conducted in the University of Leicester Cerebral Haemodynamics in Ageing and Stroke Medicine (CHiASM) research laboratory. The laboratory was quiet with minimal distractions and at a temperature between 20 and 24 °C. TCD ultrasound (Viasys Companion III; Viasys Healthcare, Beckton Dickinson & Co, Franklin Lakes, NJ) was used to measure CBv in the middle cerebral artery (MCAv) in the dominant hemisphere using a 2-MHz probe attached to a head-frame. One study also measured MCAv in the non-dominant hemisphere [8], but the two other studies recorded CBv in the non-dominant posterior cerebral artery (PCAv) [10, 18]. The cerebral vessels were located through the temporal window based on their depth, velocity and waveform. EtCO_2_ was continuously monitored using nasal prongs (Salter labs), by an infra-red capnograph (Capnocheck Plus; Smiths Medical, Ashford, UK). A three-lead electrocardiogram (ECG) was used to measure heart rate (HR). A Finometer monitor (Finapres Medical Systems, Amsterdam, The Netherlands), attached to the middle finger on the left hand was used to take continuous blood pressure (BP) recordings throughout the protocol. In addition, BP recordings from a brachial sphygmomanometer (OMRON Model 705IT; Omron Corp., Kyoto, Japan), were taken prior or at the start of each recording. The Finometer was allowed to calibrate between recordings and the servo-correcting feature was turned off during each recording. All data channels were recorded continuously using a data acquisition system (PHYSIDAS, Department of Medical Physics, University Hospitals of Leicester, Leicester, UK) at 500 samples per second.

### Continuous estimates of dynamic cerebral autoregulation (dCA)

All data editing and analyses were performed in Fortran running in Microsoft DOS on a laptop with an Intel i5- 2520 M CPU at 2.5 GHz. Data were inspected visually to check their quality and calibrated to the recorded brachial systolic and diastolic BP. Narrow spikes (< 100 ms) were removed using linear interpolation and the MCAv recordings were then passed through a median filter using offline software previously developed by the CHiASM group. All signals were then low-pass filtered with a zero-phase Butterworth filter with a 20 Hz cutoff frequency. Automatic detection of the QRS complex of the ECG, to mark the R–R interval was used, but also visual inspection was undertaken with manual correction whenever necessary. MAP, HR, EtCO_2_ and mean MCAv were calculated for each cardiac cycle, spline interpolated, and resampled at 5 Hz to generate time series with a uniform time-base, independently of fluctuations in HR. Only MCAv from the dominant hemisphere was included in the study.

Estimates of ARI for the baseline period were calculated with classical transfer function analysis using the stable poikilocapnic phase of both recordings of each individual [24, 25]. From the gain and phase spectra of the combined cross-spectra from the two distinct recordings, the impulse response of the MAP-MCAv relationship was obtained with the inverse fast Fourier transform and then integrated along time to obtain the MCAv step response, which, compared to the 10 template step responses proposed by Tiecks et al. [4], led to the resulting value of ARI. As described previously, ARI estimates were only accepted for recordings with coherence above the 95% confidence limit and fitting of the MCAv step response to the Tiecks templates with a normalised mean-square error less than 0.30 [26].

To be able to express the influence of PaCO_2_ on dCA, it is necessary to estimate its efficiency as a function of time, to parallel the corresponding changes in MCAv that take place in response to hypercapnia and hypocapnia. For time-based parameters that can represent dCA, we have chosen the ARI, proposed by Tiecks et al., due to its widespread application in both physiological and clinical studies [4, 27, 28]. To each value of ARI, ranging from 0 to 9, it is associated an MCAv response to a step change in MAP, given by a second order differential Eq [4]. It is possible to demonstrate, by means of the Z-transform [7], that the second order differential equation proposed by Tiecks et al. (4) can be expressed as an autoregressive model with exogenous inputs (ARX) structure (27), that is:

1 $$v(n) = ap(n) + b\left[ {p(n - 1) - v(n - 1)} \right] + c\left[ {p(n - 2) - v(n - 2)} \right]$$

Where *v(n)* represents time-varying values of MCAv and *p(n)* corresponding values of MAP. To improve the quality of estimates, the ‘whiteness’ of the MCAv and MAP time-series was increased by sub-sampling to 0.6 s, that is using one of each three samples [27]. [*a*,* b*,*c*] are real-value coefficients that are obtained by least-squares, for a window of 60 s duration. By means of a step change in MAP, Eq. [1] was then used to generate a step response for MCAv that could be fitted to one of the 10 template responses proposed by Tiecks et al. [4], to obtain an estimate of ARI for the 60 s window. By shifting the 60 s window for the duration of one sample (0.6 s), and repeating the procedure, it was then possible to obtain time-varying estimates of *ARI(n)* for the entire six minute recordings.

From a dynamic perspective, the influence of PaCO_2_ on *MCAv(n)* and *ARI(n)* involves a time delay of the order of 5–10 s [7, 29]. To minimize the influence of this delay on the identification of the LCM, temporal values of EtCO_2_, *MCAv(n)* and *ARI(n)* were averaged every 30 s, leading to a total of 24 samples available for estimation of the LCM by combining both recordings. Each sample corresponds to data pairs [*x*_*i*_,*y*_*i*_] where *x*_*i*_ are averaged values of EtCO_2_ for i = 1, 2, …24 and *y*_*i*_ are the corresponding values of either *MCAv(n)* or *ARI(n)*.

### The logistic curve model

The LCM is given by the classical equation:

2 $$\:y={y}_{max\:}+\:\frac{{y}_{min}-\:{y}_{max}}{1+\:{e}^{k\left(x-{x}_{0}\right)}}$$

**Fig. 1:**
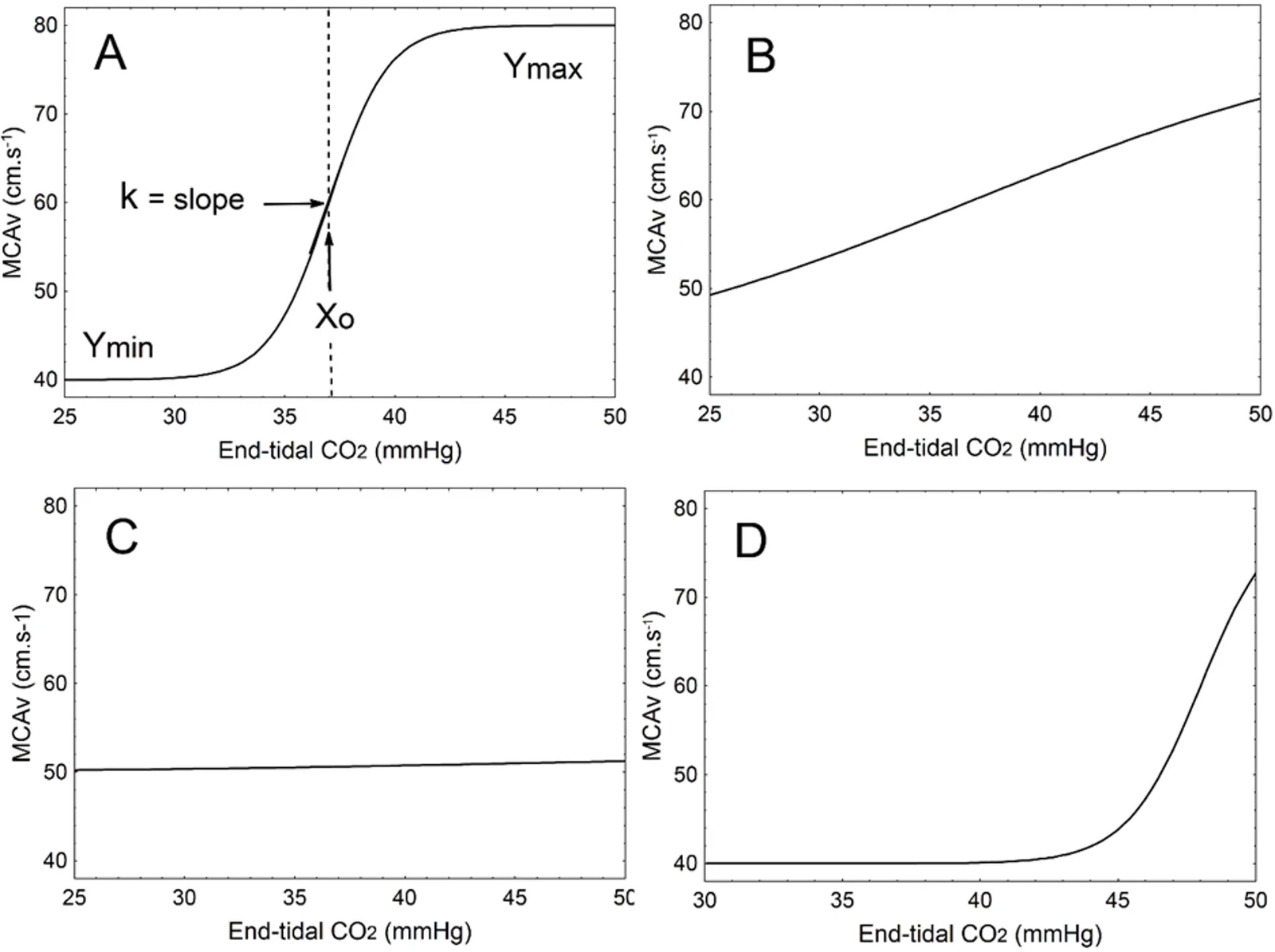
Typical logistic curve model (LCM) (**A**) and variations (**B**-**D**) due to different parameters Y_min_, Y_max_, k and X_o_ (**A**) (see Methods). (**A**) Y_min_=40 cm.s^− 1^, Y_max_=80 cm.s-^1^, k = 0.75 mmHg^− 1^, X_o_=37 mmHg; (**B**) LCM resembling a linear relationship when k is reduced to 0.1 mmHg^− 1^; (**C**) LCM as a nearly constant value with a small difference between Y_max_ (52 cm.s^− 1^) and Y_min_ (50 cm.s^− 1^), hence an almost absent CO_2_ VMR; and (**D**) absence of saturation for hypercapnia, due to a shift of X_o_ to 48 mmHg

Where *x* corresponds to values of EtCO_2_ and *y* represents the parameter being modelled (e.g. MCAv), with extreme values y_min_ and y_max_. X_0_ is the value of EtCO_2_ at the maximum first derivative (d*y*/d*x*), with slope *k*, which can be seen as akin to a ‘gain’ parameter, reflecting the rate of transition from y_min_ to y_max_. Figure 1.A illustrates the typical logistic curve, including the parameters of Eq. [2]. Figures 1.B-D represent variations of the typical curve, resulting from different parameter values.

Automated identification of parameters *y*_*min*_, *y*_*max*_, *k* and *X*_*0*_ involves three different stages:

### Estimation of parameters for a fixed number of samples (N_samp_)

As described above, a maximum of 24 data pairs [*y*_*i*_, *x*_*i*_] (*i* = 1,2…24) are available for all subjects but as explained below, with the removal of outliers, only *N*_*samp*_≤ 24 samples might be used to construct the LCM. Initial estimates of *y*_*min*_ and *y*_*max*_ are obtained as:

3 $$\:{y}_{min}=\frac{1}{3}\sum\:_{j=1}^{3}{y}_{j}$$

4 $$\:{y}_{max}=\frac{1}{3}\sum\:_{j=N-2}^{N}{y}_{j}$$

Where *y*_*j*_ are values of *y*_*i*_ ranked by *x*_*i*_ and N=*N*_*samp*_.

- With N samples, Eq. [2] is fitted to the data by identifying optimal values of *X*_*0*_ and *k*, for the initial estimates of [*y*_*min*_, *y*_*max*_] that minimize the mean square error (*MSE*), given by:

5 $$\:\epsilon^{2}=\frac{1}{N}\sum\:_{j=1}^{N}{\left(y\left({x}_{j}\right)-{y}_{j}\right)}^{2}$$

where ε^2^ is the *MSE*, *y(x*_*j*_*)* are the values of *y* from Eq. 2 and *y*_*j*_ are the data values.

b) Minimization of MSE was performed with exhaustive search in a 1000 × 1000 matrix of *k* and *X*_*0*_ values for *k* ranging from zero to 2.0 mmHg^-1^ and *X*_*0*_ ranging from 25 to 50 mmHg.

### Gradual elimination of outliers and calculation of MSE = f(N_samp_)

After the *MSE* is calculated for all *N*_*samp*_ available, this algorithm identifies the sample that, if removed, would contribute to the largest reduction in *MSE*. For the purposes of this process, this sample is referred to as an *outlier*, which is then removed, thus reducing the value of *N*_*samp*_ by one. Elimination of outliers is an important stage of the process of robust identification of LCMs mainly due to the high variability resulting from the estimation of *ARI(n)*, as will be discussed later.

**Fig. 2:**
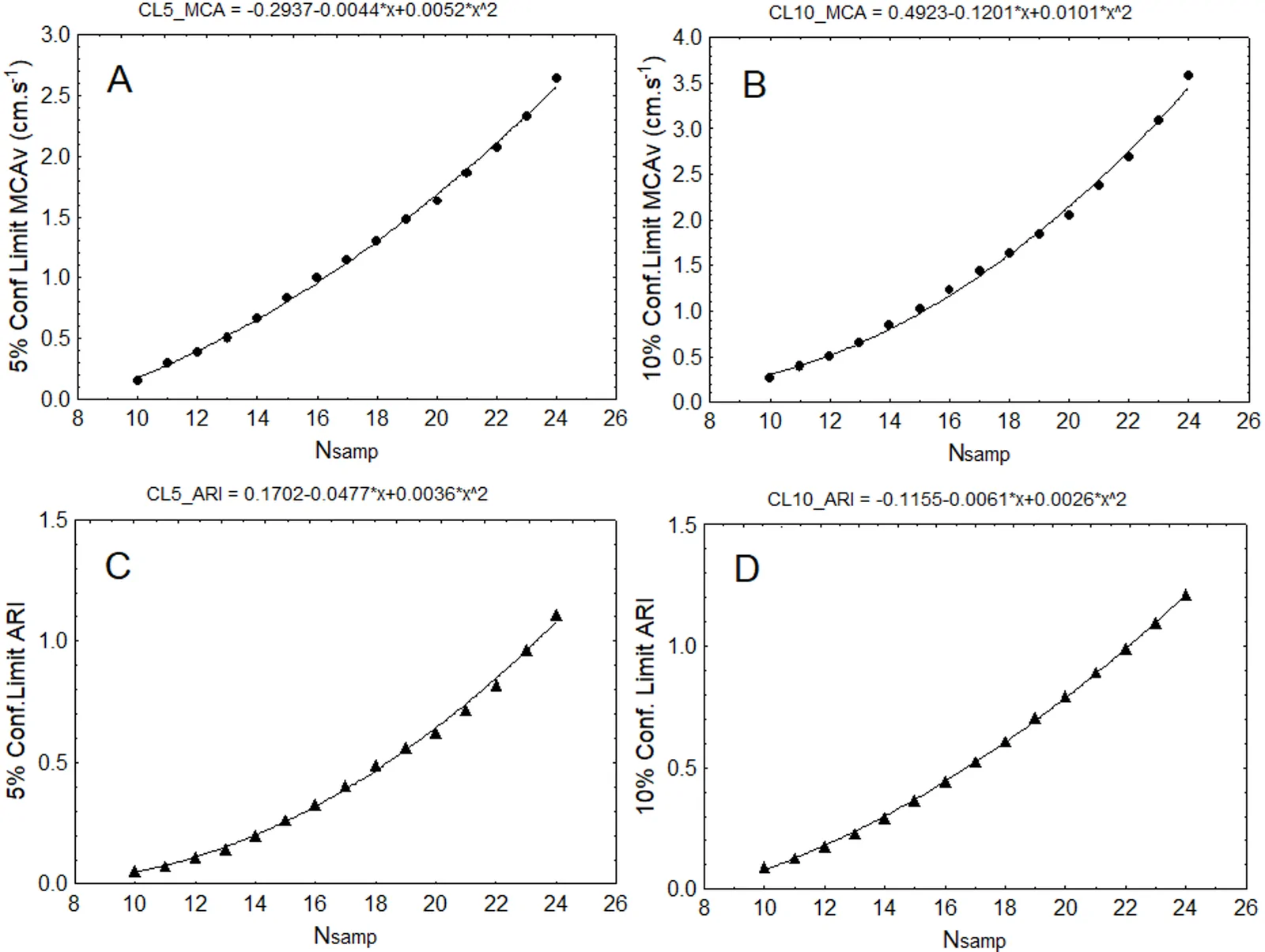
Confidence limits of the mean square error of fitting the logistic curve model (LCM) for *N*_*samp*_ number of data points based on 105 × 106 permutations of surrogate data files from 106 healthy subjects. The continuous line corresponds to the 2nd order polynomial interpolation. (**A**) 5% confidence limits for MCAv; (**B**) 10% confidence limits for MCAv; (**C**) 5% confidence limits for ARI; and (**D**) 10% confidence limits for ARI. The corresponding interpolating polynomials are given above each plot, with *x* corresponding to end-tidal CO_2_ in mmHg

This process is repeated iteratively until *N*_*samp*_ reaches a minimum pre-established value of 10 samples, thus producing 24 − 10 + 1 = 15 values of *MSE* that, by definition will be a monotonically increasing function of *N*_*samp*_ (Fig. 2).

### Estimation of confidence limits for the null hypothesis

The null hypothesis (H_0_) is that neither MCAv or ARI will have a significant dependence from EtCO_2_ following a LCM. To obtain the distribution of MSE for H_0_, we used real data measurements from 106 healthy subjects, with measurements as described in 2.1 and 2.2, and performed two combined transformations, to randomize the MCAv and ARI values, thus destroying their dependence on EtCO_2_. Firstly, we generated a surrogate data set by swapping the independent variable (i.e. EtCO_2_) of one subject with the dependent variable (i.e. MCAv or ARI) of all other subjects, thus generating 105 × 106 = 11,130 surrogate data sets. Secondly, we randomized the sequence of values of the dependent variable for each of the 11,130 data sets. Using the procedures described in 2.3.1 and 2.3.2, we then fitted the LCM to obtain the H_0_ distribution of MSE values and from these estimated the 5% and 10% confidence limits. As shown in Fig. 2, the confidence limits are very well fitted by a parabolic relationship with *N*_*samp*_ for both the MCAv and ARI models.

### Optimization of the residual number of samples

**Fig. 3:**
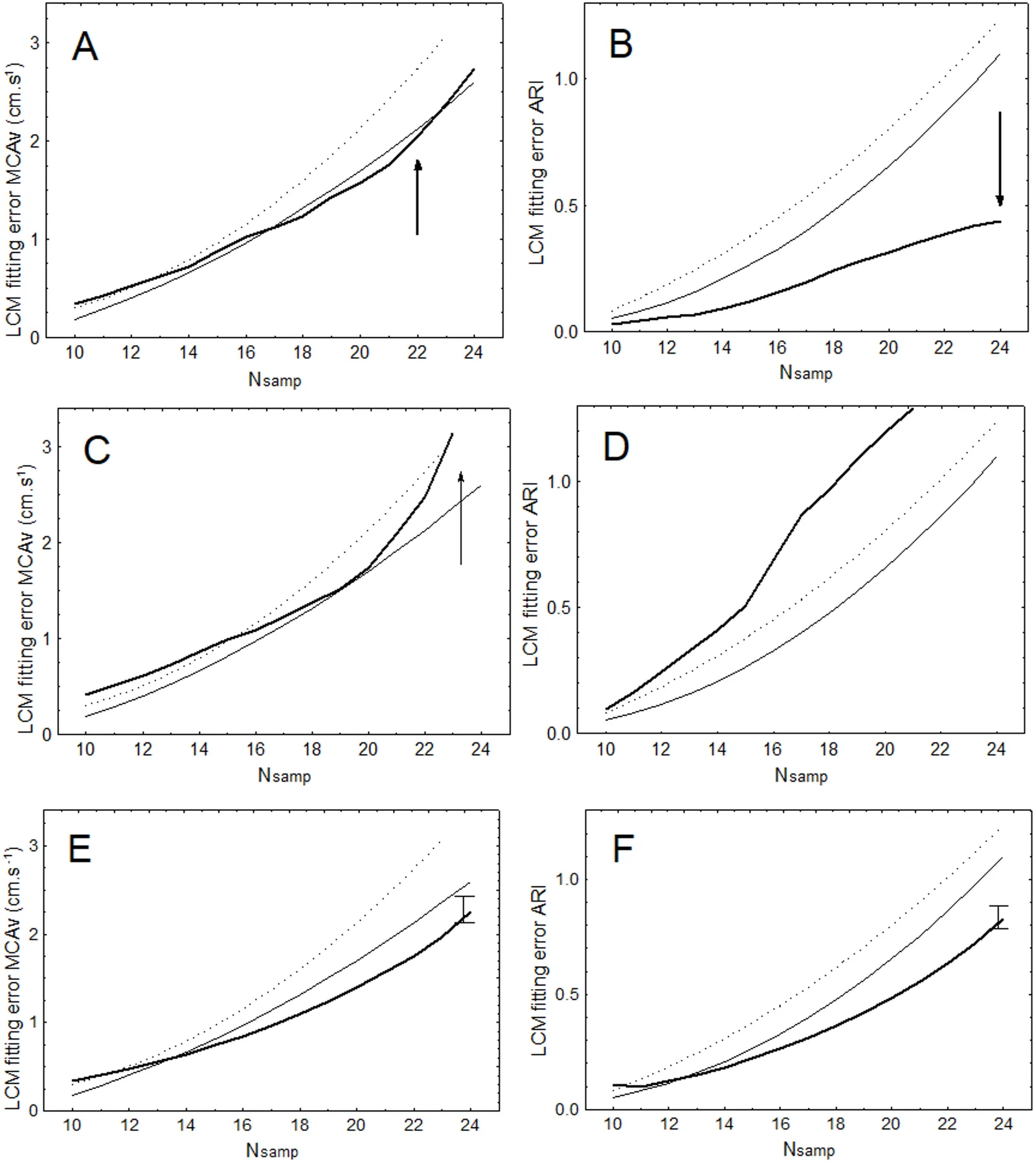
Representative curves from individual subjects (**A**-**D**) and population average (E, F) fitting errors for the logistic curve model (LCM) as a function of the number of data points (*N*_*samp*_) for MCAv (A, C,E) and ARI (B, D,F) models. The vertical arrows (**A**-**C**) indicate the *N*_*opt*_ number of samples for the optimization strategy based on maximizing the number of samples. In each plot, the thin lines correspond to the 5% (continuous line) and 10% (dashed line) confidence limits (see Fig. 2) whilst the thick continuous line represents the fitting error curve for individual subjects or population averages. (**A**) Representative error curve for a subject providing *N*_*opt*_=22 number of samples at the 5% confidence limit for the MCAv model; (**B**) Representative subject for the case of an error curve that is below the 5% confidence limit for all values of *N*_*samp*_, resulting in *N*_*opt*_=24; (**C**) In this subject, the error curve for the MCAv model is never below the 5% confidence limit, but *N*_*opt*_*=23* was found at the 10% confidence limit; and (**D**) one of the 11 subjects that were rejected for the ARI model, since the error curve was always above the 10% confidence limit. For the population averages (**E**, **F**), the error bars correspond to the largest ± 1 SE at the point of occurrence

As indicated by Fig. 3, the MSE = f(*N*_*samp*_) curves can yield a range of *N*_*samp*_ values that meet the 5% or 10% confidence limits requirement to reject H_0_ and provide reliable LCM parameters. To consistently identify optimal values of *N*_*samp*_ (*N*_*opt*_), we adopted an error minimization strategy. Starting with *N*_*samp*_=24, this algorithm identified the maximum number of samples that met the 5% confidence limit criterion, and, if not possible, then the 10% confidence limit. If neither of these conditions could be met, then the subject was excluded from further analyses.

In addition to the statistical procedures described above, normality of the data was tested with the Shapiro-Wilk W statistic and differences between parameters were assessed with the dependent or independent t-test or the Mann-Whitney U test. Values of *p* < 0.05 were considered significant.

## Results

The three previous studies yielded a total of 106 good quality recordings, corresponding to 52 female and 54 male subjects, with ages ranging from 19 to 74 years. In agreement with well-known differences due to biological sex [30], females had higher MCAv and HR but lower EtCO_2_, compared to males, without differences in age, MAP or ARI (Table 1).

**Table 1:** Demographic characteristics and main physiological parameters

Parameter	All subjects (*n* = 106)	Female (*n* = 52)	Male (*n* = 54)	*P*-value
Age (years)	34.7 ± 13.4	34.4 ± 12.8	35.1 ± 14.0	0.80
MAP (mmHg)	87.7 ± 10.4	86.7 ± 10.7	88.7 ± 10.2	0.32
MCAv (cm.s^− 1^)	60.4 ± 14.1	64.4 ± 14.6	56.6 ± 12.6	**0.004**
EtCO_2_ (mmHg)	38.9 ± 5.5	37.7 ± 5.6	39.9 ± 5.3	**0.039**
HR (bpm)	68.2 ± 10.8	70.4 ± 10.1	66.1 ± 11.2	**0.049**
ARI	4.69 ± 1.79	4.62 ± 1.81	4.76 ± 1.78	0.70

Values are mean ± SD. MAP: mean arterial blood pressure; MCAv: middle cerebral artery blood velocity; EtCO_2_: end-tidal CO_2_; HR: heart rate; ARI: Autoregulation Index. P-values for independent t-test for differences between males and females with values of *p* < 0.05 in bold

Construction of two LCM models (MCAv and ARI), for each of the 106 subjects, on a laptop adapted to run Fortran under Microsoft DOS, took approximately 15 min, corresponding to 4.24 s per LCM.

### Confidence limits of the model fitting error

Figure 2 presents the confidence limits for the MCAv and ARI models’ fitting errors as a function of *N*_*samp*_, obtained from 11,130 surrogate combinations. For MCAv, the 5% and 10% limits, for *N*_*samp*_=24 were 2.64 and 3.58 cm.s^− 1^, respectively. For the ARI, the corresponding values were 1.11 and 1.21, for the 5% and 10% confidence limits, respectively (Fig. 2). As the algorithm gradually removes outliers (see Methods 2.3.2), confidence limits are also gradually reduced following a parabolic curve as a function of *N*_*samp*_ (Fig. 2). A second order polynomial showed an excellent fitting to the data points (Fig. 2, continuous line), with coefficients given at the top of each plot in Fig. 2.

### Model fitting error for healthy subjects

When the EtCO_2_-MCAv and EtCO_2_-ARI relationships are modelled automatically with the algorithm performing gradual elimination of outliers, the corresponding fitting error curves also show a gradual decrease with the reduction in *N*_*samp*_ from 24 down to 10 samples to construct the model (Fig. 3). Individual curves for the fitting error can take different shapes, as illustrated in Fig. 3 (A-D) and these are important to appreciate the results of the strategy to identify values of *N*_*opt*_, including those cases where fitting could only be achieved at the 10% confidence limit (Fig. 3.C), or, in a few cases, when it was not possible to fit the model, leading to the removal of the subject from the study (Fig. 3.D). As indicated by the population average curves however, for the ensemble as a whole, the fitting error curves led to a majority of cases meeting the 5% confidence limit criterion, for both MCAv and ARI models (Fig. 3.E-F).

### Optimal parameter estimates

For the entire population (*n* = 106), eight subjects were rejected for the MCAv models (five females) and 11 subjects were rejected for the ARI LCM (six females). For the 98 subjects accepted in the MCAv LCM, 88 cases met the 5% confidence limit condition, with the other 10 being accepted at the 10% level. The corresponding values for the ARI models were 83 cases at the 5% level and another 12 meeting the 10% confidence level condition. In further analyses, all subjects accepted at either confidence level were pooled, thus corresponding to 98 subjects for the MCAv LCM and 95 subjects for the ARI models.

**Fig. 4:**
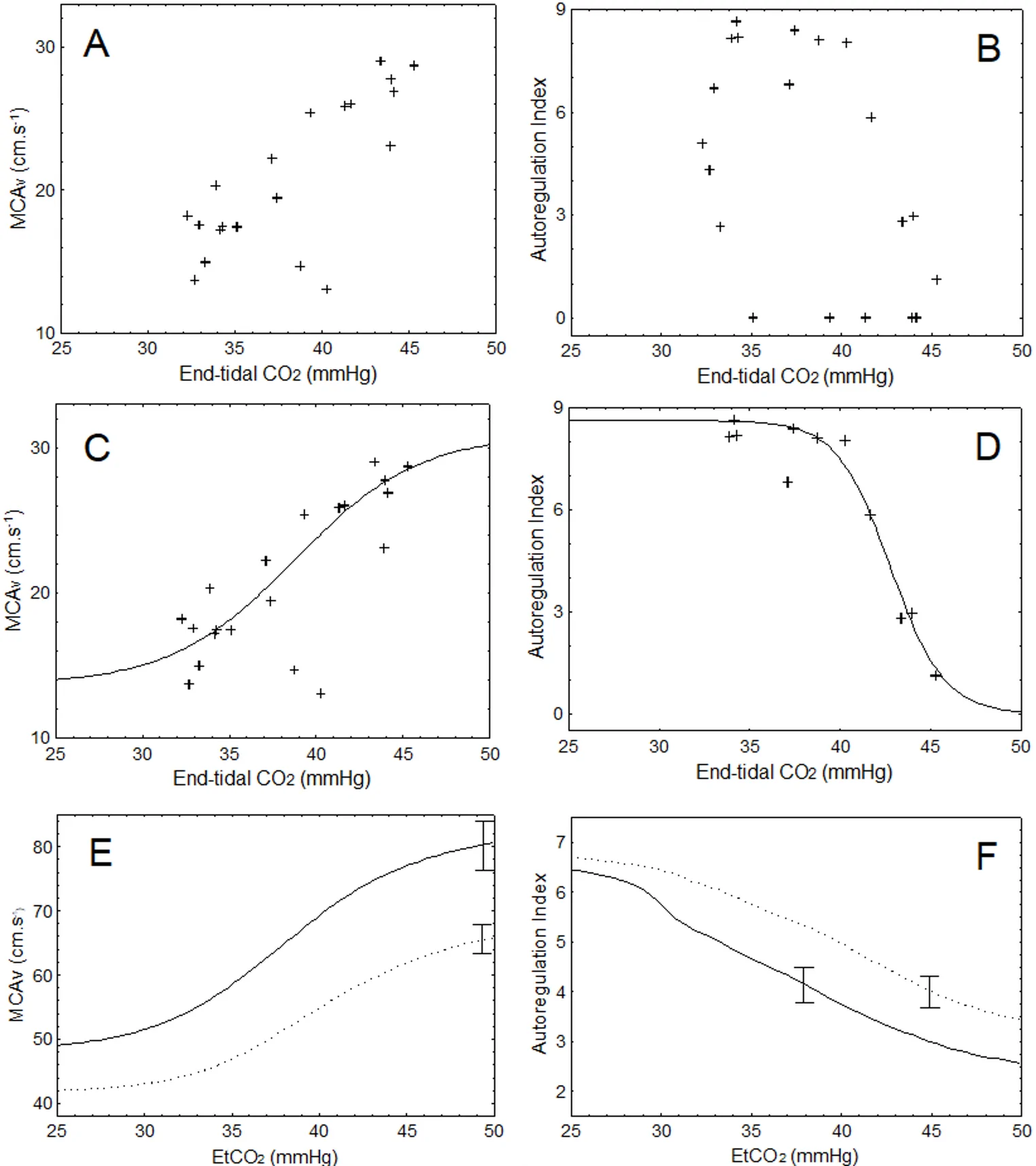
Representative example of fitting the LCM for a 26 year old female subject (**A**-**D**) and population average curves (**E**) for 98 subjects who met the 5% (*n* = 88), or 10% (*n* = 10) confidence limit conditions for the MCAv LCM and (**F**) for 95 subjects who met the 5% (*n* = 83), or 10% (*n* = 12) confidence limit conditions for the ARI LCM. Individual original scatter diagrams (*N*_*samp*_=24) for MCAv (A, MSE = 1.7 cm.s^− 1^) and ARI (**B**, MSE = 1.6). (**C**) After optimization the MCAv LCM was fitted with *N*_*opt*_=24 samples without a reduction in MSE; (**D**) corresponding result for ARI with *N*_*opt*_=15 samples and MSE reduced to 0.26. (**E**) MCAv population LCM for female (continuous line) and male (dashed line) subjects; (**F**) ARI population LCM for female (continuous line) and male (dashed line) subjects. The population curves in (**E**) and (**F**) were obtained from the average of all individual LCM curves. Error bars represent the largest ± 1 SEM at the point of occurrence

With the exception of *N*_*opt*_, all other parameters followed a normal distribution (Tables 2 and 3). Figure 4 illustrates the most usual situation of *N*_*opt*_=24 for the MCAv model (Fig. 4.A) and the elimination of nine outliers for the ARI model (Fig. 4.B) with *N*_*opt*_=15, with the corresponding model fittings in Figs. 4.C. and 4.D. The average population LCMs are depicted in Figs. 4.E and 4.F. For the MCAv models, the MSE was reduced from 2.25 ± 1.05 to 1.86 ± 0.60 cm.s^− 1^ without any differences between females and males (Table 2, *p* = 0.17). Most models had *N*_*opt*_=24 samples (Table 2). For MCAv models, only 10 subjects had *N*_*opt*_<24 and only one subject had *N*_*opt*_=11. Optimization of LCMs for ARI, with the maximum number of samples, was also dominated by *N*_*opt*_=24 samples, with 13 subjects having *N*_*opt*_<24 and only one having *N*_*opt*_=10 (Table 3). MSE for ARI models was minimally reduced from 0.83 ± 0.37 to 0.80 ± 0.25, also without differences due to biological sex (Table 3, *p* = 0.42).

**Table 2:** Distribution of MCAv LCM coefficients for all accepted subjects and separated by sex

Parameter	All accepted subjects (*n* = 98)	Females (*n* = 47)	Males (*n* = 51)	*P*-value
EtCO_2min_ (mmHg)	28.8 ± 5.9	27.9 ± 6.1	29.6 ± 5.6	0.17
EtCO_2max_ (mmHg)	47.4 ± 5.8	46.8 ± 5.9	48.0 ± 5.7	0.31
MCAv_min_ (cm.s^− 1^)	44.7 ± 9.9	47.8 ± 11.3	41.8 ± 7.3	**0.002**
MCAv_max_ (cm.s^− 1^)	75.1 ± 17.8	82.7 ± 19.3	68.0 ± 13.0	**< 0.001**
k (mmHg-1)	0.41 ± 0.21	0.39 ± 0.20	0.43 ± 0.22	0.44
X_0_ (mmHg)	39.62 ± 4.3	38.3 ± 4.6	40.1 ± 3.9	**0.035**
MSE (cm.s^− 1^)	1.86 ± 0.60	2.02 ± 0.62	1.73 ± 0.56	**0.017**
N_opt_	24 [24,24]	24 [24,24]	24 [24,24]	0.61

Values are mean ± SD or median [lower, upper quartiles]. EtCO_2min_: minimum value of end-tidal CO_2_ (EtCO_2_) for each subject; EtCO_2max_: maximum value of EtCO_2_ for each subject; MCAv_min_: minimum value of middle cerebral artery blood velocity (y_min_ in Eq. [2]); MCAv_max_: maximum value of middle cerebral artery blood velocity (y_max_ in Eq. [2]); k: gain coefficient in Eq. [2]; X_0_: EtCO_2_ value at the peak derivative of Eq. [2]; MSE: mean least square error for the optimal fitting of Eq. [2]; N_opt_: optimal number of samples for MSE algorithm (see Methods 2.3.4). P-values for independent t-test or Mann-Whitney U test for differences between female and male subjects. P-values < 0.05 are in bold

**Table 3:** Distribution of ARI LCM coefficients for all accepted subjects and separated by sex

Parameter	All accepted subjects (*n* = 95)	Females (*n* = 46)	Males (*n* = 49)	*P*-value
EtCO_2min_ (mmHg)	28.4 ± 6.1	27.9 ± 6.2	28.8 ± 6.1	0.46
EtCO_2max_ (mmHg)	47.6 ± 5.7	47.2 ± 6.0	48.1 ± 5.5	0.45
ARI_max_	7.10 ± 1.00	7.21 ± 1.01	7.00 ± 0.98	0.29
ARI_min_	2.52 ± 1.89	2.23 ± 1.85	2.80 ± 1.90	0.15
k (mmHg-1)	0.69 ± 0.73	0.78 ± 0.76	0.61 ± 0.70	0.27
X_0_ (mmHg)	38.2 ± 6.7	36.3 ± 6.2	40.1 ± 5.7	**0.0053**
MSE (cm.s^− 1^)	0.80 ± 0.25	0.82 ± 0.25	0.78 ± 0.25	0.42
N_opt_	24 [24,24]	24 [24,24]	24 [24,24]	0.61

Values are mean ± SD or median [lower, upper quartiles]. EtCO_2min_: minimum value of end-tidal CO_2_ (EtCO_2_) for each subject; EtCO_2max_: maximum value of EtCO_2_ for each subject; ARI_min_: minimum value of Autoregulation Index (ARI) (y_min_ in Eq. [2]); ARI_max_: maximum value of ARI (y_max_ in Eq. [2]); k: gain coefficient in Eq. [2]; X_0_: EtCO_2_ value at the peak derivative of Eq. [2]; MSE: mean least square error for the optimal fitting of Eq. [2]; N_opt_: optimal number of samples for MSE algorithm (see Methods 2.3.4). P-values for independent t-test or Mann-Whitney U test for differences between female and male subjects. P-values < 0.05 are in bold

The effectiveness of the maneuvers leading to changes in EtCO_2_ was demonstrated by the change from *EtCO*_*2min*_ to *EtCO*_*2max*_ (Table 2, *p* < 0.001), which was larger in the rejected subjects for the MCAv LCM (*n* = 8, *EtCO*_*2min*_ *p* = 0.01; *EtCO*_*2max*_; *p* = 0.036).

Population LCMs, calculated from the mean coefficient values in Tables 2 and 3, reflected differences due to biological sex already noticed at baseline (Table 1). For females, the LCM of MCAv was above the corresponding curve for males, for the entire range of EtCO_2_ (Fig. 4.E), as confirmed by the highly significant differences in Table 2, corresponding to *p* = 0.001 for *MCAv*_*min*_ and *p* < 0.001 for *MCAv*_*max*_. Moreover, comparison of the extreme values, suggests that the LCM of females is not a simple upward shift of the entire curve, as the difference at *MCAv*_*max*_ (14.7 ± 16.4 cm.s^− 1^) is larger than the corresponding difference at *MCAv*_*min*_ (6.1 ± 9.5 cm.s^− 1^, p = < 0.001). For the ARI, despite the visual impression of a higher *ARI*_*max*_ in females, compared to males, this was not significant (Table 3, *p* = 0.15). On the other hand, the lower baseline EtCO_2_ observed in females, compared to males (Table 1), was expressed on their LCM, showing a horizontal shift to the left, as indicated by the significant differences in *X*_*0*_ (Table 3, *p* = 0.0053). Finally, the *k* parameter (Tables 2 and 3) was higher for the ARI LCM, when compared to the corresponding values for MCAv (*p* < 0.001).

## Discussion

### Originality and contribution

In previous communications, we described the estimation of the LCM, based on the algorithm for gradual removal of outliers, with the use of visual inspection and operator-dependent decision-making for identifying *N*_*opt*_ [8, 10, 18]. This study has led to significant improvements, by removing the need for operator involvement and introducing a procedure based on rigorous estimation of confidence limits for the MSE as a function of *N*_*samp*_. To our knowledge, a similar approach for identification of the LCM of CO_2_ VMR has not been reported before.

LCM parameter values obtained with the automatic procedure, could be compared with corresponding values obtained previously with the operator-dependent approach. As shown in Table 4, although there are differences between individual studies, as indicated by mean values of parameters, inspection of their dispersion, as shown by the standard deviations, demonstrate considerable superposition of the distributions. The same considerations apply when comparing values of Ymin, Ymax, k and X_0_ from these individual studies with corresponding values generated by the automatic method (Tables 2 and 3).

**Table 4:** Summary of previous studies reporting LCM parameters

Study	Variable	*n*	Y_min_	Y_max_	K (mmHg^− 1^)	X_0_ (mmHg)
Minhas et al. [8]	MCAv	45	41.2 ± 9.3	70.5 ± 19.2	0.40 ± 0.20	36.5 ± 3.6
	ARI_MCA_	45	2.9 ± 1.9	6.9 ± 1.0	0.30 ± 0.20	36.3 ± 4.9
Clough et al. [10]	MCAv	22	46.5 ± 15.4	75.1 ± 27.8	0.60 ± 0.25	36.3 ± 2.7
	ARI_MCA_	22	2.68 ± 1.27	7.25 ± 0.70	0.84 ± 0.71	36.6 ± 4.8
Alshehri [18]	MCAv	53	43.4 ± 10.3	71.2 ± 17.4	0.52 ± 0.40	40.4 ± 5.4
	ARI_MCA_	53	2.05 ± 1.49	7.25 ± 0.84	0.67 ± 0.66	39.7 ± 7.26

Values for combined male and female subjects. For MCAv, units of Y_min_ and Y_max_ are cm.s^− 1^

In addition to removing the subjectivity of operator-dependent decision-making, the automated method led to a considerable reduction of the data processing time, thus allowing its application in large-scale studies, such as clinical trials, and paving the way towards future implementation in bedside devices.

### Methodological considerations

From the perspective of simply fitting a curve to data pairs, by means of polynomial or logistic regression, our method does not add anything to the extensive literature already available. The justification for its need though, lies with the nature of the problem involved, that is, to provide a more comprehensive and robust quantification of CO_2_ VMR in healthy subjects, as well as the complete description of the influence of PaCO_2_ on dynamic CA. For this purpose, the most common approach has been the use of TCD for measurements of MCAv or PCAv, accompanied by capnography, or other techniques, for estimation of PaCO_2_ [1]. As with any physiological measurement, recordings of MCAv, MAP and EtCO_2_ are subject to noise and drift, which contribute to the appearance of outliers in the EtCO_2_-MCAv relationship. Even more critical, is the attempt to model the EtCO_2_-ARI relationship. In baseline conditions, with stable poikilocapnia, it is possible to obtain estimates of the ARI for measurements lasting several minutes and, with the benefit of the Welch method, to reduce bias and coefficient of variation in transfer function analysis, to estimate a single value of ARI, typically for recordings lasting five minutes or more (Table 1) [24, 25]. However, this approach is not possible with much shorter time intervals containing changes in PaCO_2_, induced by either 5% CO_2_ in air or hyperventilation, when there is a need to have concomitant changes in ARI as a function of time. For this purpose, different time-domain methods could be considered for deriving *ARI(t)*, such as the moving-window ARX structure that we adopted in this study, or the orthogonal decomposition approach pioneered by Korenberg [31, 32]. We are not aware of any other reports with alternative methods for expressing *ARI(t)*. However, other metrics of dynamic CA, such as the phase difference between MAP and MCAv have been modelled with the Kalman filter [33, 34], multimodal pressure-flow modelling [35], or cross-correlation of the MAP-MCAv signals [36, 37]. Other markers of dynamic CA, such as the Mx flow index [38], or the COx index, obtained with near-infrared spectroscopy, have been reported as time-varying descriptions of autoregulation by means of a moving Pearson’s correlation coefficient [39]. Wavelet analysis has been increasingly used in studies of dynamic CA [40, 41], to generate a measure of synchronization between MAP and MCAv, that can be regarded as a marker of dynamic CA efficiency as a function of time [42]. In principle, these or any other methods that can generate estimates of dynamic CA efficiency as a function of time, with a temporal resolution of 30 s or less, could be easily adapted to provide the LCM of MCAv or other surrogates of CBF, and dynamic CA, using the same framework that we describe in this study. For this purpose though, it is key to acquire data reflecting both hypercapnia and hypocapnia in each subject. Both Liu et al. and Kostaglou et al. reported time-varying changes in phase for a step change in PaCO_2_ [33, 34], but, to our knowledge, corresponding changes in response to hypocapnia, in the same individuals, have not been described with other methods, apart from the *ARI(t)*, as mentioned above [7, 32].

Most LCMs for MCAv and ARI had *N*_*opt*_=24, corresponding to 89.8% and 86.3%, respectively, of model estimates, and it could then be argued that the automated removal of outliers is a superfluous feature of our approach. However, without this algorithm, another 10 subjects would need to be rejected for the MCAv models and, for the ARI LCM, another 13 participants. Moreover, with other datasets, that could contain a higher number of artifacts, or worse signal-to-noise ratio, as it is often the case in critical care environments, the number of cases where the MSE below the 5% or 10% confidence limits could not be achieved with the original total number of *N*_*samp*_=24 could be much higher and, without the automated removal of outliers, a much higher rejection rate would ensue.

### Physiological and clinical relevance

The LCM can be regarded as a new paradigm for the comprehensive representation of CO_2_ VMR and dynamic CA that could provide substantial advantages to both physiological and clinical studies, compared to most approaches that have hitherto been proposed. With physiological studies, uninterpretable results could be obtained if the effects of PaCO_2_ on cerebral hemodynamics are not taken into consideration, for example, resulting from changes in posture, exercise or autonomic nervous system activity. Even when EtCO_2_ is measured, and considered in the interpretation of results, corresponding changes in MCAv or CA indices might not necessarily imply that significant changes have taken place in regulatory mechanisms, as it could simply be the result of the operating point moving along the same logistic curve (Fig. 4.E or 4.F). Only when the LCM shows horizontal or vertical shifts, or a combination of these, it would be possible to say that substantial changes in the underlying physiology have taken place, for example an enhancement of CO_2_ VMR or a depression of dynamic CA. This new approach to the interpretation of physiological studies, where PaCO_2_ plays a relevant role, is exemplified by the study from Clough et al., showing that changes in posture led to lateral and vertical shifts of the LCM for MCAv, PCAv, MAP and the ARI [10].

Biological sex is increasingly regarded as a key determinant of physiological function [30], and its influence on cerebral hemodynamics is well expressed by the population differences we found in this study, with major differences in the LCM for MCAv and ARI (Tables 2 and 3; Figs. 4.E and 4.F). The higher values of CBF and MCAv in females, when compared to males is well known, but we are not aware of previous reports describing the larger differences between females and males in hypercapnia, when compared to hypocapnia (Fig. 4.E, *p* = 0.0031). These findings reinforce the need for biological sex to be included as a key determinant of CBF regulation in any future studies of cerebral hemodynamics [43].

Similarly to physiological studies, clinical assessments of CO_2_ VMR and dynamic CA could lead to erroneous findings if the entire range of EtCO_2_ is not considered, as expressed by the LCM of MCAv and ARI. As an example, very different results would be obtained with VMR markers, depending on the operating point on the LCM. The dominant protocol for assessment of VMR is to induce hypercapnia and express VMR as the ratio (MCAv^after^ – MCAv^before^)/(EtCO_2_^after^-EtCO_2_^before^), where *before* and *after* refer to normocapnia and hypercapnia, respectively. Considering the shape of the LCM in Fig. 4.E, it is clear that much smaller values of VMR will be obtained as the operating point at normocapnia (EtCO_2_^before^) moves to the right. In fact, this is often the situation with some clinical conditions where hypocapnia is prevalent, thus implying a lower EtCO_2_ operating point at rest [44, 45]. For this reason, obtaining the LCM in clinical populations should be regarded as a priority to determine if the hypercapnia or hypocapnia observed in certain disease conditions, such as ischemic stroke or chronic obstructive pulmonary disease (COPD) [44, 45], simply reflect a different operating point along the curve, or a shift of the entire LCM resulting from the underlying pathology. When differences in EtCO_2_, between patients and control groups, are detected in cross-sectional comparative studies, these are usually adjusted with multivariate linear methods, that do not take into consideration the correct physiological dependence of MCAv, or other hemodynamic parameters, on PaCO_2_. This limitation could be overcome with the use of the LCM for this purpose. Until reference LCM curves can be obtained for ischemic stroke and other conditions, it is not possible to align the EtCO_2_ operating point of patients towards the corresponding value of healthy individuals, but this should not stop us from making the inverse adjustments, from health to disease, using the LCM obtained in this study. As an example, if a male patient with a stroke has an ARI = 4 with EtCO_2_=36 mmHg, and the control group has the same value of ARI at EtCO_2_=40 mmHg, the difference will not be statistically significant. However, after adjusting the ARI of controls to the same EtCO_2_ of 36 mmHg, with Eq. [2] and parameters in Table 3, the resulting value of ARI for controls would be approximately ARI = 7, therefore clearly showing the relative depression of dynamic CA in the patient with a stroke.

To be able to roll out the application of the LCM approach to clinical studies though, more work is needed to acquire enough data to allow characterization of the influence of age on LCM parameters, particularly in females, considering how menopause can influence CBF regulation [46, 47]. Paramount would be the exploration of how the LCM can be influenced by different disease conditions, not only for its diagnostic and prognostic possibilities, but also for its potential to bring additional knowledge of the effects of pathology on CBF regulation.

### Limitations of the study

The relationship between TCD measurements of MCAv and absolute CBF is dependent on the MCA cross-sectional area, which has been shown to be altered at extreme values of PaCO_2_ [48, 49]. For this reason, it would be prudent to assume that with increases in MCA diameter at *EtCO*_*2max*_, and reductions in diameter at *EtCO*_*2min*_, that the corresponding values of *MCAv*_*max*_ and *MCAv*_*min*_ in Table 2 are, respectively, underestimates and overestimates of their true values. If this is the case, then the true LCM would show a larger difference between the maximum and minimum velocities, compared to what is represented in Table 2; Fig. 4.E. Important to note though, that errors in the measurement of MCAv amplitude do not influence corresponding values of ARI or *ARI(t)* as these are only dependent on the temporal pattern of the MCAv step response [4, 26].

We obtained estimates of the LCM model parameters (*Ymax*, *Ymin*, *k*, *X*_*0*_) using exhaustive search of a 1000 × 1000 partition of each coefficient. Despite its limited computational efficiency, we preferred this option for its robustness, considering that the automated method was designed to run on occasional data analyses, not as an everyday task. Future work could consider alternative algorithms for the identification of model parameters that could improve speed and accuracy.

A major assumption of this study is that the LCM, that has been suggested to describe the CBF dependence on PaCO_2_ [17, 19–21], can be extended to also represent the way the ARI and other cerebrovascular parameters are influenced by PaCO_2_. Our assumption is based on a combination of theoretical and pragmatic considerations. Firstly, under stable physiological conditions, or even in the presence of pathology compatible with life, most biological variables show the phenomenon of saturation at both extremes, and this is a key feature of the LCM. Secondly, the ‘logistic law’ has been shown to be pervasive in biology, including chemical reactions, population growth and epidemic contagion and, hence, would be a prime candidate to represent the non-linear dependence of cerebrovascular parameters on PaCO_2_, when considering its entire physiological range. Finally, despite having only four parameters (Eq. [2]), the LCM can accommodate different ‘shapes’ as illustrated in Fig. 1 for MCAv. By inverting maximum and minimum values, similar diversity of curves would be obtained for the ARI. However, these arguments should not deflect further studies aimed to refine the quantitative relationship between PaCO_2_ and CBF, ARI, or other parameters. To do so in human studies though, is likely to require a much larger volume of data and it would be important to also consider the cost-benefit between what is to be gained in model accuracy, compared to the additional complexity of handling a much larger number of parameters that these models will likely demand.

 [31, 32].

Finally, we have made some arbitrary choices that would benefit from more in-depth scrutiny. For averaging the MCAv and ARI(t) time-series, we have chosen a duration of 30 s that generated a maximum of N_samp_=24 points for construction of the LCM. We have also chosen a minimum number of 10 averaged points to be considered in the estimation of confidence levels, or for eliminating outliers. Extensive sensitivity analyses, beyond the scope of this initial communication, would be needed to assess the influence of these empirical choices on the robustness of LCM.

## Conclusions

In a relatively large cohort of healthy subjects, we have demonstrated that it is possible to identify the LCM for the effects of PaCO_2_ on MCAv and ARI, with an automated algorithm based on the confidence limits of the model fitting error. In females, the LCM for MCAv is always above the corresponding curve for males, but with relatively higher values at hypercapnia when compared to hypocapnia. For the ARI LCM, the curve for females shows a lateral shift to the left thus helping to explain the usual finding of lower values of EtCO_2_ at rest, compared to men. Further work is needed to characterize the influence of aging on the LCM of healthy individuals and to extend a similar approach to clinical populations.

## Data Availability

Data available upon reasonable request to corresponding author.

## Abbreviations

ABP: Arterial blood pressure
ARI: Autoregulation index
ARX: Autoregressive model with exogenous inputs
CA: Cerebral autoregulation
CBF: Cerebral blood flow
CBv: Cerebral blood velocity
CHiASM: Cerebral hemodynamics in age and stroke medicine
COPD: Chronic obstructive pulmonary disease
CVR: Cerebrovascular resistance
ECG: Electrocardiogram
EtCO_2_: End-tidal carbon dioxide
HR: Heart rate
LCM: Logistic curve model
MAP: Mean arterial blood pressure
MCAv: Middle cerebral artery blood velocity
MSE: Mean square error
Mx: Mean flow index
N_opt_: Optimal number of samples
N_samp_: Number of model samples
PaCO_2_: Partial pressure of arterial carbon dioxide
PCAv: Posterior cerebral artery blood velocity
PHYSIDAS: Data acquisition system
TCD: Transcranial Doppler
VMR: Vasomotor reactivity to carbon dioxide
